# Composite Cement Materials Based on β-Tricalcium Phosphate, Calcium Sulfate, and a Mixture of Polyvinyl Alcohol and Polyvinylpyrrolidone Intended for Osteanagenesis

**DOI:** 10.3390/polym15010210

**Published:** 2022-12-31

**Authors:** Kseniya Stepanova, Daria Lytkina, Rustam Sadykov, Kseniya Shalygina, Toir Khojazoda, Rashidjon Mahmadbegov, Irina Kurzina

**Affiliations:** 1 Faculty of Chemistry, Tomsk State University, 634050 Tomsk, Russia; 2 Faculty of Natural Science, Russian-Tajik (Slavonic) University, Dushanbe 734000, Tajikistan

**Keywords:** bone cement, composite, β-TCP, PVA, PVP

## Abstract

The primary purpose of the study, presented in this article, was to obtain a composite cement material intended for osteanagenesis. The β-tricalcium phosphate powder (β-TCP, β-Ca_3_(PO_4_)_2_) was obtained by the liquid-phase method. Setting and hardening of the cement system were achieved by adding calcium sulfate hemihydrate (CSH, CaSO_4_·1/2H_2_O). An aqueous solution of polyvinyl alcohol (PVA), polyvinylpyrrolidone (PVP), and a PVA/PVP mixture were used as a polymer component. The methods of capillary viscometry and Fourier-transform infrared spectroscopy (FTIR) revealed the formation of intermolecular hydrogen bonds between polymer components, which determines the good miscibility of polymers. The physicochemical properties of the synthesized materials were characterized by X-ray diffraction (XRD) and FTIR methods, and the added amount of polymers does not significantly influence the processes of phase formation and crystallization of the system. The size of crystallites CSD remained in the range of 32–36 nm, regardless of the ratio of polymer components. The influence of the composition of composites on their solubility was investigated. In view of the lower solubility of pure β-TCP, as compared to calcium sulfate dihydrate (CSD, CaSO_4_·2H_2_O), the solubility of composite materials is determined to a greater degree by the CSD solubility. Complexometric titration showed that the interaction between PVA and PVP impeded the diffusion of calcium ions, and at a ratio of PVA to PVP of 1/1, the smallest exit of calcium ions from the system is observed. The cytotoxicity analysis results allowed us to establish the fact that the viability of human macrophages in the presence of the samples varied from 80% to 125% as compared to the control.

## 1. Introduction

The development of new biomaterials for bone tissue restoration is a relevant topic in the field of regenerative medicine [1]. This trend is conditioned by the prevalence of bone tissue defects resulting from fractures, infectious and tumor processes, age-related osteoporosis, and some other pathological conditions of the bone. Bone tissue regeneration is a limited and time-consuming process; therefore, it is necessary to use bone substitutes [2]. In addition, serious disadvantages of natural bone grafts are known: An autograft requires additional surgical interventions and does not exclude the probability of donor site morbidity, and an allograft can cause tissue rejection and transmit infections [3].

Bone cement can be defined as a family of materials consisting of powder and liquid phases that, after mixing, form a plastic paste that can self-cure once implanted into the body. This means that the material is moldable, which ensures a perfect fit to the implant site and good bone-to-material contact, even in geometrically complex defects [4]. Recent advances in orthopedic surgery are associated with the use of minimally invasive surgical techniques. In this area, it is critical to have injectable materials available, and in this sense, bone cement can play a decisive role, provided that injectable cement is developed. An example is the performance of some minimally invasive surgical procedures, namely vertebroplasty and kyphoplasty, to repair vertebral compression fractures, in which bone cement is injected into the vertebral body [4].

Improving the properties of bone cement can be achieved by reinforcing the cement matrix with either particles or fibers [5,6,7,8,9,10]. In some cases, the addition of water-soluble polymers can be used to change the adhesion of the cement slurry or its rheological properties. For construction cement, there is evidence that the combined use of sulfates and polyvinyl alcohol adversely affects the strength of cement [11]. However, for bone cement, there are studies confirming that the addition of certain water-soluble polymers can be very effective in improving the adhesion of bone cement pastes [12,13].

Of the many biomaterials, synthetic calcium phosphates (CaP) have proven themselves in a number of studies because of their chemical similarity to the bone mineral component [14,15]. CaP is used in the form of ceramic products [16,17], coatings on metal implants [18], or in the form of cement pastes [19,20]. Bone cement is characterized by a paste-like consistency, set at the body temperature, which allows molding the implant at the site of the defect with a firm adherence to the surrounding tissues, as well as using a minimally invasive technique of a surgical operation by means of injection [21,22,23,24,25,26].

Among CaP, hydroxyapatite (HA, Ca_10_(PO_4_)_6_(OH)_2_) and tricalcium phosphate (TCP, Ca_3_(PO_4_)_2_) have become preferable for bone tissue regeneration [3,27,28,29,30]. Based on published results [15,31], β-TCP tends to have higher osteoconductivity as compared to that of HA and α-TCP. HA has been reported to behave similarly to an inert implant [32], while β-TCP is moderately dissolved as a result of cell-mediated resorption [33,34,35,36,37]. The main disadvantage of α-TCP is that the resorption rate is too fast [38]. The research results in [39] show that β-TCP is an excellent material with respect to stem cell differentiation and in vivo osteoinduction compared to pure HA and an HA/TCP mixture.

We selected a β-TCP-based material for the study. The cement powder was obtained by adding calcium sulfate (CS), which has been used as an orthopedic biomaterial for many years [40,41,42]. In [43], the effect of the CS/β-TCP composite was studied as compared to that of β-TCP with the example of treating iliac bone defects occurring in dogs. Four months later, CS/β-TCP demonstrated a much greater contribution to the new bone formation as opposed to that of pure β-TCP. The addition of medical gypsum will allow for obtaining a material with selective resorption, where the calcium-phosphate matrix acts as a relatively stable framework and CS acts as a pore-former and setting component. The inclusion of biocompatible polymers in the cement formulation will allow the most complete imitation of the natural matrix of the bone tissue and improve the mechanical properties of the material. Polyvinyl alcohol (PVA) and polyvinylpyrrolidone (PVP) are suitable biodegradable and nontoxic polymers [44,45], and a mixture based on them improves the in vivo stability of polymers, that is, it allows for preventing the PVA suspension formation under physiological conditions [30].

## 2. Materials and Methods

β-TCP was synthesized by the liquid-phase method at a pH of 11 according to the following equations:

9Ca(NO_3_)_2_ + 6(NH_4_)_2_HPO_4_ + 6NH_4_OH → Ca_9_(HPO_4_)(PO_4_)_5_OH + 18NH_4_NO_3_

Ca_9_(HPO_4_)(PO_4_)_5_OH → 3β-Ca_3_(PO_4_)_2_ + H_2_O

Solutions of calcium nitrate Ca(NO3)2 1.2 M and ammonium hydrophosphate (NH_4_)_2_HPO_4_ 0.8 M were prepared separately at room temperature (25 °C). Ca(NO_3_)_2_ and (NH4)_2_HPO_4_ were purchased from Sigma-Aldrich (MO, USA). The initial reagents were taken in equimolar volumes so that the molar ratio of Ca/P elements was 3/2. The Ca(NO_3_)_2_ solution was added dropwise to the (NH_4_)_2_HPO_4_ solution while stirring on a magnetic stirrer. pH 11 was maintained by adding a concentrated ammonia solution (35%). Next, to establish the effect of mixing time on the phase composition of the phosphate, half of the suspension continued to be stirred for 12 h; in the case of the second half of the suspension, the next synthesis step immediately began. The white precipitate was washed with distilled water and isopropyl alcohol. The suspension was subjected to vacuum filtration. The formed precipitate was dried at 80 °C for a day. The dried powder was crushed and then calcined at 900 °C for two hours to form the TCP beta phase. In the first part, after mixing, all the same operations were performed.

PVA (with an average molecular weight of 104,500 g/mol) was purchased from Sigma Aldrich (Saint Louis, MO, USA). PVP (with a molecular weight of 3500 g/mol) was provided by Acros Organics (Geel, Belgium). The PVA 5 wt% solution was prepared in distilled water at 90 °C during constant mixing with a magnetic stirrer for 1 h. The aqueous PVP 5 wt% solution was prepared at room temperature during constant stirring for 15 min. The PVA/PVP 5 wt% mixture was prepared by mixing freshly prepared solutions in the ratios of 1/3, 1/1, and 3/1.

The minimum amount of medical gypsum required for setting the β-TCP-based cement was selected experimentally. For this reason, calcium sulfate hemihydrate (CSH, CaSO_4_·1/2H_2_O) was mixed in amounts of 50, 40, 30, 20, and 10 wt% with β-TCP and water in a powder-to-liquid ratio of 1/1. When adding 30 wt% of gypsum, the setting was noticeable, whereas, at 20 and 10 wt%, the consistency was still quite liquid. The following powder composition was chosen: 30 wt% of CSH and 70 wt% of β-TCP.

Composite cement materials were obtained by direct mechanical mixing of β-TCP/CSH powder components with the addition of pure water and the PVA, PVP, and PVA/PVP solutions in the ratios of 1/3, 1/1, and 3/1. The powder and liquid were mixed in a ratio of 1/1. The synthesis was carried out in Petri dishes. The setting time of the samples varied from 7 to 10 min. The obtained cement paste was left to harden completely.

The phase composition of the initial components and composites was determined using a MiniFlex 600 diffractometer (Rigaku). The survey photographing of the samples was performed under monochromatic CuKα (α = 1.5418 Å) radiation in the reflection angular range of 2θ = 5–100° in increments of 0.02°, with a voltage of 40 kV and a speed of 3 °/min. The phases were decoded and identified using the ICDD diffraction database (PDF-2/Release 2012 RDB). To calculate the coherent scattering region (CSR), the reflex value at its half-height was determined using the Gaussian function in Origin software.

The spectra of the surface layer of the samples were recorded by means of the Nicolet 6700 IR-Fourier spectrometer (Thermo Scientific). The spectra were recorded with a resolution of 4 cm−1 in the range of 400–4000 cm^−1^, using the FTIR attachment.

The 5 wt% solution viscosity of the PVA/PVP mixture, depending on the mixing time of the solutions, was measured at room temperature using a capillary viscometer (d = 1.31 mm).

To determine the solubility, the samples were soaked in the physiological solution at 37 °C for 20 days. The total weight loss of the samples and the total concentration of calcium ions in the solution were recorded every 5 days.

The weight losses were calculated as a percentage using the formula:

Losses, wt% = (mi − mк)·100%/mi

where mi is the initial weight of the sample and mк is the sample weight after soaking the sample in distilled water.

The Ca^2+^ concentration in the solution was determined by complexometric titration in the presence of Eriochrome Black T and the ammoniac buffer solution with a pH of 10.

The viability of immune system cells on the surface of the studied materials was assessed using the Alamar Blue indicator. The optical density was measured using a Tecan Infinite 200 microrider at wavelengths of 573 and 605 nm.

The data were analyzed using Student’s *t*-test and presented as an average ± standard deviation. Three duplicate samples were used in all the experiments. The data probability was considered statistically significant for values of *p* < 0.05.

## 3. Results and Discussion

**Figure 1:**
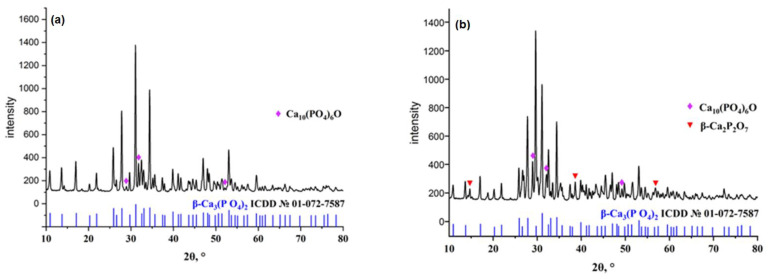
Sample left to mix for 12 h: (**a**) β-Ca_3_(PO_4_)_2_, (**b**) Ca_10_(PO_4_)_6_O.

The X-ray diffraction (XRD) measurement results allowed us to establish that the sample (Figure 1a), left to mix for 12 h, contained two phases: A major β-TCP and a hydroxyapatite (HA) admixture. At the same time, the sample (Figure 1b) obtained without mixing contained the third phase: Beta-form calcium pyrophosphate (β-Ca_2_P_2_O_7_). In this way, such mixing influences the phase formation process of calcium phosphate. The appearance of HA and β-Ca_2_P_2_O_7_ admixtures may be caused by an inhomogeneous distribution of the components in the solution during the deposition of β-Ca_3_(PO_4_)_2_ [43]. HA forms at a temperature of approximately 900 °C in air, most likely via HA dehydroxylation [46]. We selected sample (a) for further work.

**Figure 2:**
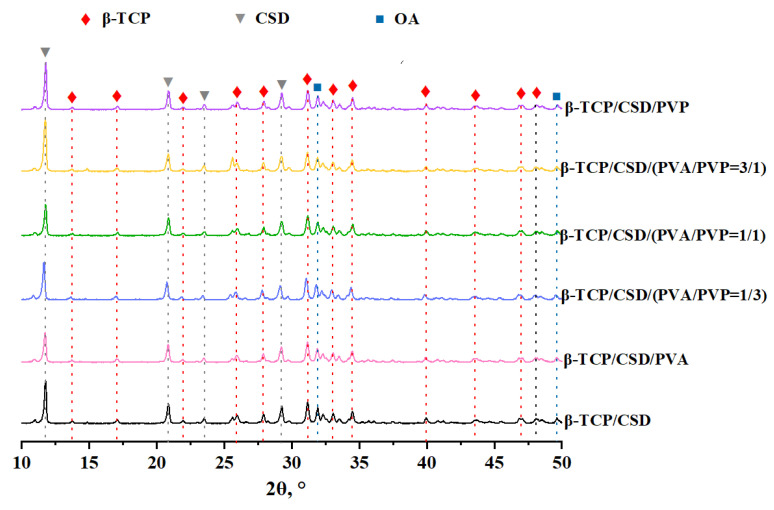
Diffraction patterns of composite cement materials.

According to the XRD data, at different ratios of the polymers, the composite samples are characterized by the same phase composition: β-Ca_3_(PO_4_)_2_, CaSO_4_·2H_2_O, Ca_10_(PO_4_)_6_O (Figure 2).

The size of the crystallites in the composite cement samples varied in the range of 28–31 nm and 32–36 nm for the β-Ca_3_(PO_4_)_2_ and CaSO_4_·2H_2_O phases, respectively (Table 1), i.e., there is a fairly uniform distribution of phases by the size of the crystallites. The CSR values for the β-Ca_3_(PO_4_)_2_ phase, included in the initial CaP and part of the composites, essentially do not change. In the case of CSH, the size of the crystallites is somewhat smaller than that of CSD included in the composites, since hydration occurs when mixing with water. Then, calcium sulfate dihydrate microcrystals grow in size, intertwine, and grow together. As a result, the addition of polymers does not significantly influence the processes of phase formation and crystallization of the system.

**Figure 3:**
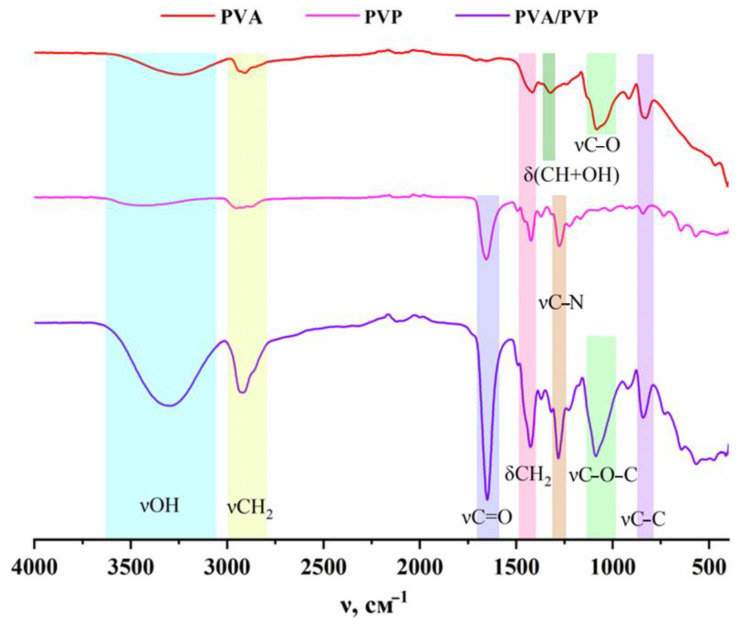
IR-spectrum of the 5% aqueous solutions of PVA, PVP, and PVA/PVP.

The Fourier-transform infrared spectroscopy (FTIR) method was used to study films of the 5% aqueous solutions of PVA, PVP, and PVA/PVP. Figure 3 shows the fundamental absorption bands of functional groups. The following specific bands were found for pure PVA [47,48]. A wide band of approximately 3343 cm^−1^ corresponds to the valence vibration of the νOH hydroxyl group. Two bands are observed at 2930 cm^−1^ and 2908 cm^−1^, corresponding to the asymmetric and symmetric valence vibrations of νCH_2_, respectively. The bands at 1418 cm^−1^ and 828 cm^−1^ are related to the scissor vibration of δCH_2_ and stretching of the zigzag-shaped carbon base of νC-C, respectively. The absorption band at 1323 cm^−1^ characterizes the deformation vibrations of δ(CH+OH). The wagging vibration of δCH is poorly observed at 1237 cm^−1^. The absorption of approximately 1084 cm^−1^ corresponds to the stretching of the acetyl groups of νC-O that are present in the main PVA chain. The weak band at 916 cm^−1^ can be referred to as the rocking vibration of δCH_2_.

The following specific bands were found for pure PVP [47,48,49,50,51]. The observed wide low-intensity band of approximately 3468 cm^−1^ is related to the stretching of the OH-group of the water adsorbed on the polymer surface. The absorption band at 2951 cm^−1^ corresponds to the asymmetrical vibration of νCH_2_. The bands at 1421 cm^−1^ and 840 cm^−1^ correspond to the scissor vibrations of δCH_2_ and the chain bending of νC-C, respectively. The bands at 1657 cm^−1^ and 1284 cm^−1^ can be referred to as valence vibrations of the νC=O carbonyl group and the stretching of the group of the νC-N pyrrolidone ring, respectively.

The participation of the hydroxyl group in the formation of intermolecular hydrogen bonds is manifested through the shift of the absorption band towards lower frequencies and a significant increase in its intensity [51]. The absorption band of the PVA hydroxyl group at 3343 cm^−1^ shifts towards lower wave numbers at 3313 cm^−1^ for the PVA/PVP mixture due to the formation of an intermolecular hydrogen bond between the PVP carbonyl group and the PVA hydroxyl group [40]. At the same time, a slight shift of the absorption band of the electron donor group by 10–20 cm^−1^ is characteristic [51]; therefore, the PVP carbonyl band group is shifted from 1656 cm^−1^ to 1649 cm^−1^.

**Figure 4:**
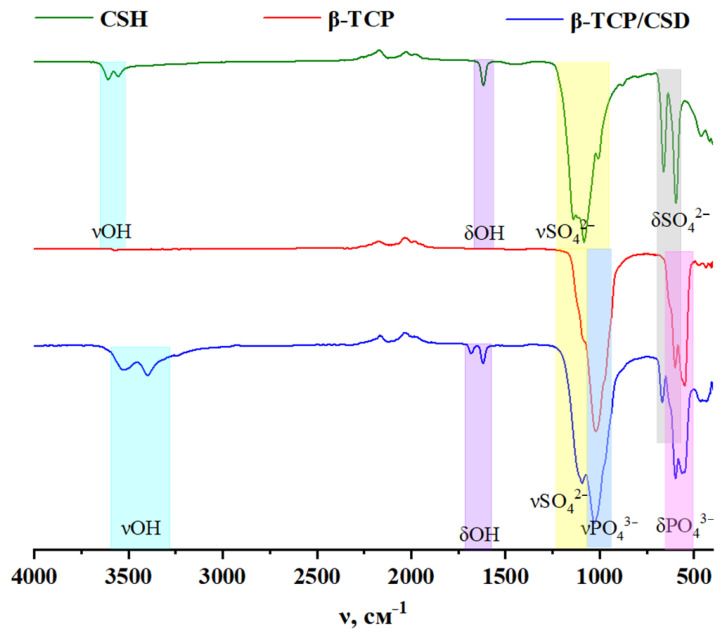
IR spectrum of CSH, β-TCP, and β-TCP/CSD water cement.

The IR spectra peculiarity of CaP is the presence of two doubled absorption bands. The first is detected at wave numbers such as ν = 900–1200 cm^−1^ and the second at ν = 350–650 cm^−1^ [52]. Figure 4 shows that the vibrations of the β-TCP phosphate groups are located at approximately 550 and 1020 cm^−1^.

In the IR spectrum of β-TCP/CSD, there is a slight shift of the bands of valence vibrations of the νPO_4_^3−^ groups to a longer wavelength region due to the formation of a new phase on phosphate particles and anion geometry distortion as a result of nonsymmetric interactions in the crystal. Specific absorption bands are also observed at 3400 cm^−1^ and 1620 cm^−1^, which are related to the adsorbed water (bound OH-group), its valence, and deformation vibrations, respectively. The band at 3527 cm^−1^ appears because of the valence vibrations of OH-ions of the crystallohydrate lattice (free OH-group) [30].

The sulfate ion can be identified by valence and deformation vibrations in the range of 980–1100 cm^−1^ and 550–650 cm^−1^, respectively [52]. In the case of CaSO_4_·1/2H_2_O, there are fluctuations in the sulfate and hydroxide ions of the lattice, as shown in Figure 4.

**Figure 5:**
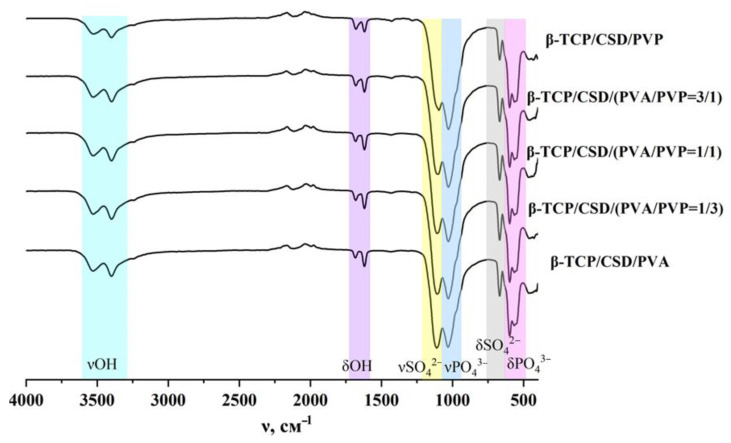
IR spectra of composite cement materials.

The IR spectra of the composites (Figure 5) differ by the small shifts of the sulfate group band, which may be related to anion geometry distortion, inductive, and mesomeric effects as a result of the interaction with polymeric chains.

**Figure 6:**
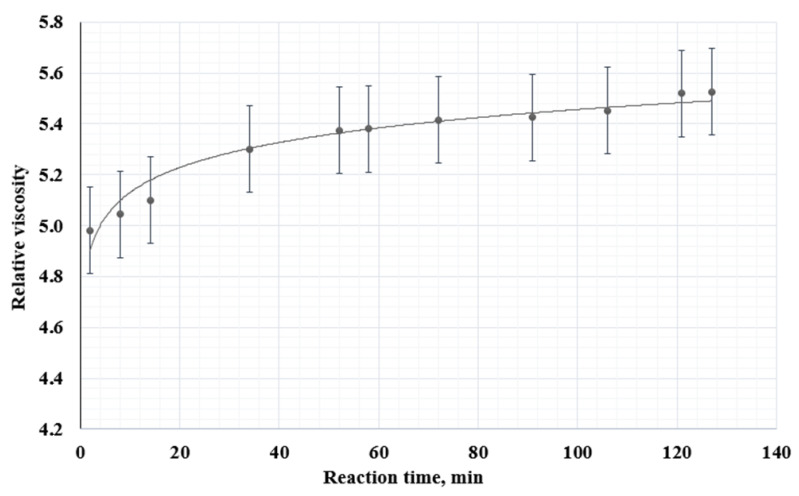
Relative viscosity measurement of the 5% solution of the PVA/PVP mixture.

A graph was constructed based on the capillary viscometry results (Figure 6). In the first 30–40 min of mixing the polymers, the relative viscosity increases significantly. These results can be explained by the interaction between PVP and PVA, conditioned by the formation of intermolecular hydrogen bonds between the carbonyl group of the pyrrolidone ring and the side hydroxyl PVA group.

A series of experiments were conducted to study the solubility of the samples. The data in Table 2 demonstrate that the weight losses of cement materials increased along with an increase in the soaking time of the samples. In view of the lower solubility of pure β-TCP as compared to CSD, the solubility of the composite materials is more determined by the CSD solubility. In the case of the composites, weight losses varied in the range of 68 to 76 wt%, while in the occasion of pure β-TCP, the losses reached only 47 wt%.

**Figure 7:**
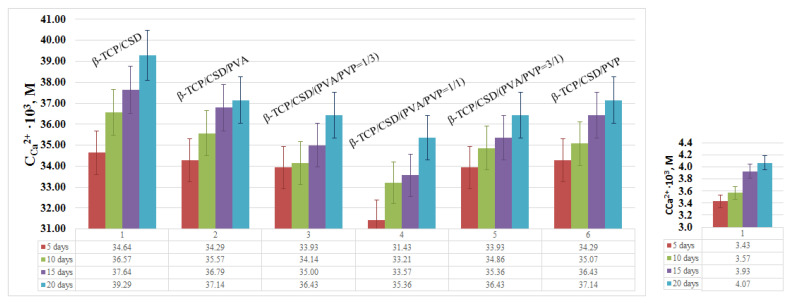
Release of Ca^2+^ ions within 20 days for the composites and the initial β-TCP.

In the case of the composites containing a mixture of polymers, the release of calcium ions is lower than that in the composites containing only one polymer (Figure 7). The interaction between PVA and PVP manifests itself in a solution viscosity increase, which hinders the diffusion of calcium ions.

**Figure 8:**
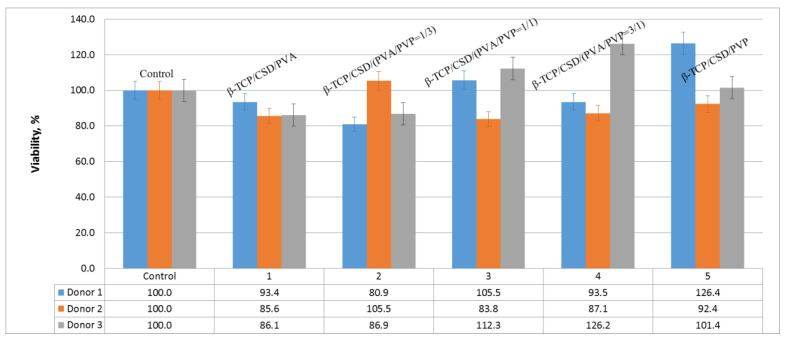
Viability of human macrophages in the presence of composite cement.

The studies conducted on human macrophages (Figure 8) have shown that, in the presence of the samples, most of the cells do not die. The viability of monocytes varies from 80% to 125% as compared to the control (100%).

Within the series, there is no unique dependence of cytotoxicity on different concentration ratios of polymers in the cement composition. Comparing the composites with individual polymers, it is possible to state that cell viability is higher in the presence of PVP, which confirms its hemocompatibility. It is also possible to observe that some donors have an individual reaction to the material: Donor 2 shows lower viability relative to other samples, which suggests that the viability of monocytes in the presence of materials is comparable to the control or higher. The fact that more cells survive in some donors than in controls indicates that the environment in the presence of materials is more favorable and more cells survive after incubation. From parallel studies [53] involving hydroxyapatite and polyvinyl alcohol-based gels, we know that PVA supplementation can significantly improve macrophage survival. In the case of bone cement, we observe the same effect. Thus, it can be assumed that the co-administration of polymers has a beneficial effect on the viability of macrophages, which shows the possibility of further development of these studies, which may consist of finding a narrower range of effective concentrations of the components, as well as establishing and improving mechanical characteristics.

## 4. Conclusions

1. In this work, a number of composite cement materials based on β-TCP and CSD were obtained and characterized with the addition of PVA and PVP solutions and a PVA/PVP mixture solution in the ratios of 1/3, 1/1, and 3/1.
2. The selected polymers mix well with each other and form a stable homogeneous solution. The formation of intermolecular hydrogen bonds between the polymer components was detected by capillary viscometry and IR-spectroscopy.
3. According to the XRD results, the added amount of polymers does not significantly influence the processes of phase formation and crystallization of the system.
4. It has been found that the solubility of the cement is determined to a greater extent by the CSD solubility in view of the lower solubility of pure β-TCP. The manifested interaction between the polymeric components hinders the diffusion and release of calcium ions.
5. The study of the cytotoxicity level of the composite cement materials has shown that most of the cells do not die in the presence of the samples. Consequently, these composites are very promising biomaterials for bone regeneration.

## Figures and Tables

**Table 1:** CSR values of cement and initial powder components.

Sample	CSR Values for Different Phases, nm		
	β-TCP	CSD	CSH
β-TCP	30	−	−
CSH	−	−	28
β-TCP/CSD	31	36	−
β-TCP/CSD/PVA	30	34	−
β-TCP/CSD/(PVA/PVP = 1/3)	29	32	−
β-TCP/CSD/(PVA/PVP = 1/1)	28	33	−
β-TCP/CSD/(PVA/PVP = 3/1)	33	36	−
β-TCP/CSD//PVP	28	32	−

**Table 2:** Weight losses of the composites and the initial β-TCP depending on the duration of sample soaking in the physiological solution.

Number of Days	Weight Losses, wt%						
	β-TCP	β-TCP/CSD	β-TCP/CSD/PVA	β-TCP/CSD/(PVA/PVP = 1/3)	β-TCP/CSD/(PVA/PVP = 1/1)	β-TCP/CSD/(PVA/PVP = 3/1)	β-TCP/CSD/PVP
5	33	34	54	63	60	47	34
10	40	48	62	65	64	60	40
15	46	67	75	67	69	72	69
20	47	68	76	70	72	76	69

## Data Availability

All data presented in this study are contained within the article.
